# Tracheostomy or Not: Prediction of Prolonged Mechanical Ventilation in Guillain–Barré Syndrome

**DOI:** 10.1007/s12028-016-0311-5

**Published:** 2016-08-18

**Authors:** Christa Walgaard, Hester F. Lingsma, Pieter A. van Doorn, Mathieu van der Jagt, Ewout W. Steyerberg, Bart C. Jacobs

**Affiliations:** 1 Department of Neurology, Room EE 2230, Erasmus Medical Center, PO Box 2040, 3000 CA Rotterdam, The Netherlands; 2 Department of Public Health, Erasmus Medical Center, Rotterdam, The Netherlands; 3 Department of Intensive Care, Erasmus Medical Center, Rotterdam, The Netherlands; 4 Department of Immunology, Erasmus Medical Center, Rotterdam, The Netherlands

**Keywords:** Guillain–Barré syndrome, Artificial respiration, Tracheostomy

## Abstract

**Background:**

Respiratory insufficiency occurs in 20 % of Guillain–Barré syndrome (GBS) patients, and the duration of mechanical ventilation (MV) ranges widely. We identified predictors of prolonged MV to guide clinical decision-making on tracheostomy.

**Methods:**

We analyzed prospectively collected data from 552 patients with GBS in the context of two clinical trials and three cohort studies in The Netherlands. Potential predictors for prolonged MV, defined as duration of ≥14 days, were considered using crosstabs. Selected predictors were analyzed using Cox regression analysis.

**Results:**

On a total of 150 (27 %) patients requiring MV, 106 (71 %) patients needed prolonged MV. The median duration of MV was 28 days (Interquartile Range [IQR] 12–60 days). The strongest observed predictors of prolonged MV were muscle weakness and axonal degeneration or unexcitable nerves on nerve conduction studies. Patients who are unable to lift the arms from the bed (bilateral Medical Research Council [MRC] of deltoid muscles of 0–2) at 1 week after intubation have an 87 % chance to require prolonged MV versus 69 % in patients who are able to lift the arms from the bed (bilateral MRC of deltoid muscles of 3–10). Patients in this last group who had axonal degeneration or unexcitable nerves on nerve conduction studies also have a 90 % chance to require prolonged MV.

**Conclusions:**

Ventilated GBS patients who are unable to lift the arms from the bed and patients who have axonal degeneration or unexcitable nerves at 1 week are at high risk of prolonged MV, and tracheostomy should be considered in these patients.

## Introduction

Respiratory failure is a life-threatening manifestation of the Guillain–Barré syndrome (GBS) that occurs in 20–30 % of patients with GBS [1–4]. Immunomodulatory treatment reduces the proportion of patients who require mechanical ventilation (MV) as well as the duration of MV [5, 6]. The duration of the required MV varies widely in GBS, ranging from a few days to several months and even longer than 1 year. In general, tracheostomy should be considered when the expected ventilation duration is more than 14 days [7, 8]. The uncertainty about the duration of required MV in individual patients may complicate this decision in clinical practice. Delayed tracheostomy in ventilated patients may result in avoidable damage of the vocal cords, laryngeal mucosa, and recurrent laryngeal nerves due to decubitus or local pressure from the endotracheal tube [7]. On the other hand, early tracheostomy may be unnecessary because of clinical improvement and exposes patients to the risk of perioperative bleeding, infection, esophageal perforation, pneumothorax, and tracheal stenosis and, in all cases, leaves a permanent scar [9].

Previous studies showed that the clinical course of GBS in individual patients can be predicted with reasonable accuracy [10–14]. In the current study, we described the variability in duration of MV and characteristics of GBS patients with prolonged MV and aimed to identify predictors of prolonged MV. These predictors may support individual clinical decision-making about indication and timing of tracheostomy in patients with GBS early in the course of their disease.

## Patients and Methods

### Patients

Prospectively collected data were used from 552 patients who fulfilled the diagnostic criteria for GBS, were treated with either plasma exchange or intravenous immunoglobulins, and did not die in the first week of hospital admission. These patients previously participated in a treatment trial [15, 16], an observational study [17], or a pilot study [18, 19] conducted by the Dutch GBS Study Group. The ethical review board of Erasmus MC approved all studies, and all patients gave written informed consent to use their data for further research.

### Data Collection

Data collected prospectively for all patients were age, gender, preceding infections, number of days from onset of weakness to hospital admission, date of intubation and extubation, and neurological examination (cranial nerve testing, sensory and motor testing; using the Medical Research Council [MRC] sumscore) at predefined time points (at admission and at 3, 7, 14, 28, 90, and 181 days after admission). The MRC sumscore is defined as the sum of MRC scores of six different muscles measured bilaterally, which results in a sumscore ranging from 0 (tetraplegic) to 60 (normal). For this study, we recorded neurological examination at 1 week after intubation. Nerve conduction studies were performed in the first 2 weeks after inclusion, and the data were used to classify GBS as acute inflammatory demyelinating polyradiculoneuropathy (AIDP), acute motor axonal neuropathy (AMAN), equivocal, or unresponsive according to the Hadden criteria [20]. Serological screening was performed to determine recent infections with *Campylobacter jejuni*, cytomegalovirus, Epstein–Barr virus, and *Mycoplasma pneumonia* and antibodies to the gangliosides GM1, GD1a, and GQ1b. The serum samples used were obtained within 4 weeks from onset of weakness and before start of treatment and were stored at −80 °C until use.

### Endpoints

The primary endpoint in our study is the occurrence of prolonged MV, defined as MV of more than 14 days, as an established criterion to consider tracheostomy [7, 8]. In addition, we determined the risk of requiring MV for more than 21 and 28 days. We defined liberation from MV as either successful extubation or spontaneous breathing off the ventilator in tracheotomized patients for more than 24 h. Predictors of prolonged MV were sought at day 7 after start of ventilation, as a clinical decision point for considering early tracheostomy. Also, we determined the time to reach the ability to walk unaided in different patient groups.

### Statistical Analysis

A Kaplan–Meier curve with log-rank test was used to compare time to reach the ability to walk unaided during a follow-up period of 6 months between patients with prolonged MV, patients with MV for <14 days, and patients not requiring MV. Potential predictors for prolonged MV were considered in crosstabs, and univariate logistic regression models and odds ratios (ORs) indicated relative effects of predictors. Cox regression analysis was used to further analyze selected predictors and calculate the estimated risk percentages for prolonged MV duration (≥14, ≥21, and ≥28 days). A Cox regression model was used since our cohort was relatively small. Using specific cut-offs of long versus short MV duration would result in low numbers of patients in specific categories and unstable models. Cox regression accounts for the total duration of ventilation and thus uses the data more efficient than logistic regression with a binary outcome (long vs. short MV). Missing values were imputed based on relevant covariates and outcome. A two-sided *p* value <0.05 was considered to be statistically significant. Statistical analyses were conducted with SPSS for Windows and *R* statistical software (version 2.7, using the design library).

## Results

### Mechanical Ventilation in GBS

**Fig. 1:**
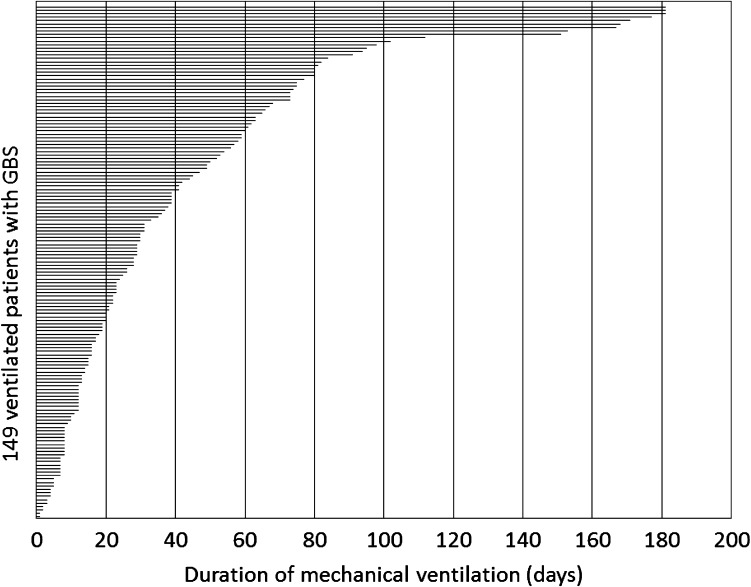
Duration of mechanical ventilation in 149 patients with GBS. The figure indicates the duration of mechanical ventilation in 149 patients with Guillain–Barré syndrome. One patient of the original cohort of 150 ventilated patients was excluded because the patient was lost to follow-up after 3 months of ventilation. Median duration of mechanical ventilation was 28 days, interquartile range of 12–60 days, absolute range 1 to >181 days (follow-up of the studies ended at 181 days)

In the cohort of 552 patients with GBS, 150 (27 %) required MV at some time during the follow-up of 6 months. The median duration of the MV was 28 days (Interquartile Range IQR 12–60 days; absolute range 1 to >81 days; Fig. 1). Patients were intubated at a median of 1 day after admission (IQR 0–4). The timing of intubation was not correlated with the MV duration (Table 1). Eight patients in the MV group (5 %) died during the follow-up period of 6 months. Mortality was not significantly different between the patients who needed prolonged MV (6 %) and those who did not (5 %). The indication for intubation was not systematically documented, but the percentage of bulbar weakness was not significantly different between the two groups.

**Table 1:** Characteristics of the cohort of 132 Guillain–Barré syndrome patients on the ventilator for at least 7 days in relation to prolonged mechanical ventilation

Characteristic	Total	*N* (%) prolonged MV (≥14 days)	Median (IQR) days on ventilator	OR (95 % CI) for prolonged MV	*p* value
Total	132	106 (80 %)	31 (16–63)		
Demographic features					
Age (in years)					NS
≤40	41	32 (78 %)	23 (16–57)	Ref	
41–60	41	34 (83 %)	40 (21–77)	1.4 (0.5–4.1)	
>60	50	40 (80 %)	30 (16–60)	1.1 (0.4–3.1)	
Gender (male)	71	57 (80 %)	30 (16–63)	1.0 (0.4–2.4)	NS
Clinical severity^a^					
Days from onset weakness to MV				1.0 (0.9–1.0)	NS
Bulbar weakness	28	22 (79 %)	52 (15–78)	0.9 (0.3–2.4)	NS
Facial weakness	56	46 (82 %)	41 (17–67)	1.2 (0.5–3.0)	NS
MRC sumscore					<0.001
41–60	18	7 (39 %)	11 (8–20)	0.2 (0.04–0.5)	
21–40	43	35 (81 %)	28 (15–44)	Ref	
0–20	71	64 (90 %)	49 (25–80)	2.1 (0.7–6.3)	
M. deltoideus^b^				0.7 (0.6–0.8)	<0.001
M. deltoideus dichotomized					0.001
MRC 0–2	61	58 (95 %)	53 (27–82)	9.3 (2.6–32.7)	
MRC 3–10	71	48 (68 %)	21 (12–40)	ref	
M. biceps^b^				0.7 (0.6–0.9)	<0.001
M. extensor carpi radialis^b^				0.8 (0.6–0.9)	0.001
M. iliopsoas^b^				0.7 (0.6–0.9)	<0.001
M. quadriceps^b^				0.7 (0.6–0.9)	<0.001
M. tibialis anterior^b^				0.8 (0.7–0.9)	0.002
Nerve conduction studies					
AIDP	64	48 (75 %)	29 (14–54)	Q	Q
AMAN	4	4 (100 %)	131 (69–178)		
Equivocal	26	20 (77 %)	23 (15–49)		
Unexcitable	15	15 (100 %)	82 (62–171)		
Infection and serology					
Symptoms of preceding infection^c^					
Diarrhea	32	27 (84 %)	45 (21–112)	1.44 (0.5–4.2)	NS
Upper respiratory tract infection	47	33 (70 %)	24 (13–66)	0.4 (0.2–0.9)	0.03
Infection serology^d^					
*Campylobacter jejuni*	43	37 (86 %)	44 (20–87)	1.8 (0.7–5.0)	NS
Cytomegalovirus	20	18 (90 %)	52 (26–63)	2.7 (0.6–12.6)	NS
Epstein–Barr virus	13	12 (92 %)	29 (23–73)	3.5 (0.4–28.1)	NS
*Mycoplasma pneumonia*	6	5 (83 %)	20 (17–26)	1.3 (0.2–11.8)	NS
Anti-ganglioside IgM/IgG antibodies					
GM1	14	12 (86 %)	91 (18–176)	1.6 (0.3–7.6)	NS
GD1a	9	8 (89 %)	23 (16–60)	2.2 (0.3–18.4)	NS
GQ1b	10	10 (100 %)	49 (16–60)	Q	Q

*MV* mechanical ventilation, *IQR* interquartile range, *OR* odds ratio, *CI* confidence interval, *MRC* Medical Research Council, *AIDP* acute inflammatory demyelinating polyradiculoneuropathy, *AMAN* acute motor axonal neuropathy. Q Because of 100 % values, it was impossible to calculate ORs and *p* values for AMAN, unexcitable nerves, and anti-GQ1b antibodies

^a^At 1 week after intubation

^b^Sum of MRC grades for bilateral muscle groups

^c^Symptoms of infection in the 4 weeks preceding the onset of weakness

^d^Using pretreatment serum samples obtained at entry

**Fig. 2:**
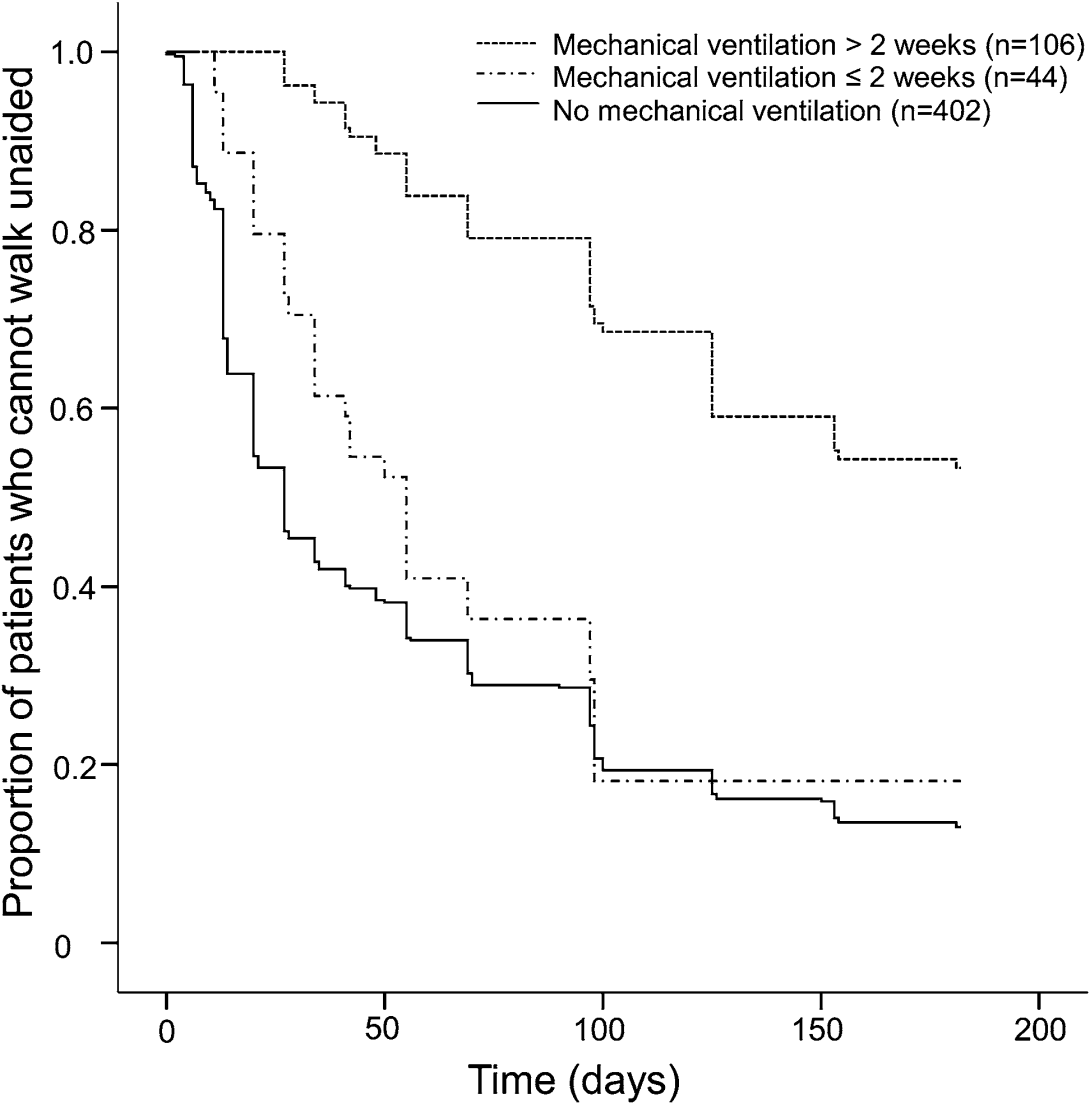
Outcome of GBS in relation to duration of mechanical ventilation. Relation between mechanical ventilation, its duration, and the time (in days) to recover to independent walking in a cohort of 552 patients with Guillain–Barré syndrome. Kaplan–Meier curves show the proportion of patients who regained the ability to walk unaided during a follow-up of 181 days

MV was associated with poor outcome, as 57 % in the ventilated group regained the ability to walk in the first 6 months, in comparison with 87 % in the unventilated group (OR 5.0 [3.2–7.7], *p* < 0.001). Patients with prolonged MV also needed more time to regain the ability to walk than the patients with MV <14 days (log-rank test, *p* < 0.001) (Fig. 2). Forty-four patients who required MV <14 days had a comparable recovery as the 402 unventilated patients (log-rank test, *p* = 0.2) (Fig. 2).

### Prediction of MV Duration

**Fig. 3:**
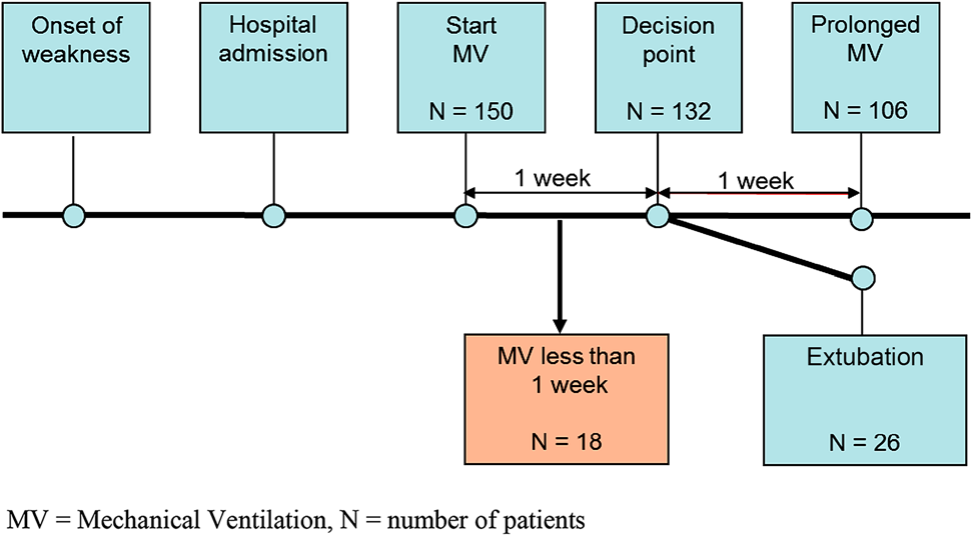
Flowchart of patient subgroups in relation to mechanical ventilation. *MV* mechanical ventilation, *N* number of patients

Predictors of prolonged MV were determined at day 7 after start of ventilation, which was considered a critical time point in clinical practice to make a decision about tracheostomy. Patients requiring MV for 7 days or less (*N* = 18) were excluded from this analysis (Fig. 3). In the remaining 132 patients, 106 (80 %) needed prolonged MV (Fig. 3). In Table 1, the observed frequencies of potential predictors and crude associations with prolonged MV are shown. The strongest predictor of prolonged MV was severe limb weakness defined by the MRC sumscore 1 week after intubation (*p* < 0.001; Table 1). Further analysis showed that the MRC scores of the bilateral deltoid muscles alone also were a strong predictor of prolonged ventilation (*p* < 0.001; Table 1). Furthermore, a total of 61 patients were unable to lift the upper arms (bilateral MRC score ≤2), and 58 (95 %) of those needed prolonged MV (OR 9.3 [2.6–32.7], *p* = 0.001; Table 1). Also, regression (OR 3.7, 95 % CI, 0.4–3.0) or improvement (OR 0.2, 95 % CI, 0.06–0.7) of muscle strength of the bilateral deltoid muscles was predictive of prolonged ventilation (*p* = 0.007). Patients with AMAN (*N* = 4) or unexcitable nerves (*N* = 15) all required prolonged MV (Table 1); and therefore, it was not possible to calculate an OR or *p* value in univariate analysis. Because of the small patient numbers in the nerve conduction study (NCS) subgroups, we divided the patients for the multivariate analysis into two groups: AMAN or unexcitable versus AIDP or equivocal. In addition, all patients with serum anti-GQ1b antibodies (*N* = 10) required MV. We used Cox regression analysis to predict chances to require MV for more than 14, 21, and 28 days in the different groups based on the condition of the patient after 1 week of mechanical ventilation (Table 2). Based on this model, patients who were unable to lift the arms from the bed had an estimated chance of prolonged MV for more than 14 days of 87 % (Table 2). NCS results significantly contribute to the prediction and can be taken into consideration.

**Table 2:** Predicted chances (Cox regression analysis) of a total mechanical ventilation duration of ≥14, ≥21, and ≥28 days

Condition at 1 week after intubation	*N*	Chance (%, 95 % CI) of a total MV duration of		
		≥14 days	≥21 days	≥28 days
Mechanical ventilation (observed)	131	80	66	55
Mechanical ventilation *and* unable to lift arms	61	87 (81–92)	77 (70–85)	68 (60–78)
Mechanical ventilation *and* unable to lift arms *and* axonal NCS/unexcitable NCS	16	96 (93–98)	93 (87–97)	89 (82–95)
Mechanical ventilation *but* able to lift arms *and* axonal NCS/unexcitable NCS	5	90 (84–96)	83 (73–93)	75 (63–89)
Mechanical ventilation *but* able to lift arms *and* AIDP or equivocal NCS	65	67 (58–78)	48 (38–60)	34 (24–46)

*AIDP* acute inflammatory demyelinating polyneuropathy, *CI* confidence interval, *MV* mechanical ventilation, *N* number, *NCS* nerve conduction study

## Discussion

The decision for tracheostomy in patients with GBS depends on the expected duration of respiratory failure, which may range from a few days to more than 6 months. In the current study, MV was required in 27 % of patients and 71 % of these patients required MV for more than 14 days. Eighty percent of the patients who were still intubated after 1 week required prolonged MV. The chance of prolonged MV was further increased in the subgroup of patients with severe paresis of the deltoid muscles, defined as being unable to lift the arms from the bed (MRC grade 0 or 1 bilaterally), and the patients with an axonal or unresponsive polyneuropathy in the NCS. In these patients, it may be considered to perform an early tracheostomy.

Our study confirms previous findings by others that the duration of MV in GBS is associated with the extent of limb muscle weakness. Fourrier et al. [14] reported that the lack of foot flexion at the end of immunotherapy was a predictor for prolonged MV in a group of 40 ventilated GBS patients in a retrospective, single-center study. They did not report on the predictive value of paresis of other limb muscles. In the current study, we confirmed the association between prolonged MV and paresis of the anterior tibial muscle; but stronger associations were found for paresis of the deltoid, biceps, iliopsoas, and quadriceps muscles. These results provide further support for the hypothesis that patients with prolonged MV have a severe diffuse neuropathy, which affects both the respiratory and limb muscles. We preferred to use the deltoid muscle for the prognostic model because of the strong association with prolonged MV, its common C5 innervation with the phrenic nerve, and relatively easy accessibility for physical examination in bed-bound patients. However, when the examination of the deltoid muscles is not possible in an individual patient, substitution of other preferably proximal muscle groups, such as the iliopsoas muscles, seems plausible.

In the current study, all ventilated patients with AMAN (*N* = 4) and unexcitable nerves (*N* = 15) required prolonged MV. This finding is in line with previous findings [1, 21]. One study indicated that the presence of AIDP was associated with a higher chance of respiratory failure [22], but we were unable to confirm that finding. The electrophysiology results are influenced by the applied classification criteria and the timing of the NCS. In Western countries, the axonal forms of GBS are relatively rare compared to AIDP and are found in 5–10 % of GBS patients. In addition, NCS performed at 1 week is less accurate for identifying axonal GBS, as the axonal pattern may appear only after 2–4 weeks. At 1 week of admission, patients more frequently show unexcitable nerves in NCS. These patients may have either AIDP or axonal forms, but in all cases this is a sign of severe diffuse neuropathy. As such, unexcitable nerves may be a more frequent indication than AMAN for early tracheostomy.

Remarkably, our study showed that all 10 ventilated patients with serum anti-GQ1b antibodies required prolonged MV. Antibodies to GQ1b in patients with GBS are strongly associated with the occurrence of ophthalmoplegia and swallowing disorders. Some studies indicated that these patients are prone to develop respiratory failure [23], but this was not found by others [11, 24]. As far as we know, the current study is the first to demonstrate the relation between GQ1b antibodies and the duration of MV in ventilated patients with GBS. Some of these patients also had severe weakness of arms and legs or unexcitable nerves, indicating that the presence of anti-GQ1b antibodies is probably not an independent prognostic factor. A further limitation of this biomarker for supporting the decision of tracheostomy in current clinical practice is the delay and quality of the test results, which are influenced by the used assay protocol. Because of these limitations, we have not used the test in the current prognostic models.

We were unable to confirm the finding from a previous retrospective, single-center study in 60 ventilated GBS patients that age is an independent predictor for prolonged MV [21]. In most studies, older age is a predominant prognostic factor for poor outcome in GBS, including those of our own group. In the current study, we found no association between age and prolonged MV (OR 1.1 for age >60 years), neither did other previous studies. Also the presence of a preceding *C. jejuni* infection, which is a general poor prognostic factor in GBS, was not predictive for prolonged MV. Previously, we showed that selective gut decontamination may shorten the time of MV and admission to ICU but does not shorten the time to reach the ability to walk [25]. Apparently, the recovery from respiratory failure depends on other factors than the recovery of limb weakness.

The current study has several limitations that need to be addressed. First, the group of ventilated patients was too small to be able to develop and validate a prognostic model, as was done previously for predicting respiratory failure in the first week in GBS [11]. To overcome this limitation in part, we used Cox regression analysis that also takes the total duration of MV into account. This resulted in slightly lower, but presumably more realistic, predictions of MV duration. For example, we observed that all patients with the axonal subtype or unexcitable nerves (100 %) needed prolonged MV. Model-based prediction in this subgroup resulted in a 93 % chance of prolonged MV (univariate; data not shown). Hence, some predictors of prolonged MV were present in even smaller subgroups, such as the axonal subtype and anti-GQ1b antibodies, although all these patients had prolonged MV. Second, the patient population investigated was biased toward adult patients and patients with AIDP, which is the predominant GBS subtype in The Netherlands. The observed finding at present cannot be extrapolated to pediatric GBS or countries where axonal forms predominate. Third, in this multicenter study, differences in the duration of MV may reflect variation in local clinical management; intubation or extubation criteria were not used in our patient group. Also, usage and timing of tracheostomy was not recorded in our cohort, and this probably influenced the duration of MV. All patients were included in previous trials, which have the advantage of protocoled, repeated clinical assessments, but could have influenced the standard clinical care. Fourth, in the current study we have used data collected in various previous studies conducted in the last 25 years. We cannot exclude that the criteria for extubation and supportive care have changed over time. In the future, international prospective validation studies in larger cohorts of GBS patients, including children and patients from other regions and with clear definitions regarding extubation criteria, will be needed to substantiate our findings.

Debate is still ongoing about the optimal timing of tracheostomy. A consensus report on MV indicated that the translaryngeal route is preferred when the expected duration is not exceeding 10 days, while tracheostomy is preferred for expected durations longer than 21 days [26]. Prolonged MV via the translaryngeal route carries significant risks, while tracheostomy has its own complications and leaves permanent disfigurement. Nowadays prospective trials show that early tracheostomy was associated with less sedative and analgesic administration, less frequent prescriptions of haloperidol to treat agitation or delirium, earlier oral nutrition, and out-of-bed mobilization. Early tracheostomy does not seem to shorten the duration of MV, length of hospital stay, mortality, or frequency of infectious complications [8, 27–30].

## Conclusion

Most GBS patients on MV after 1 week will require prolonged MV, and the chances are further increased in patients with severe deltoid muscle weakness and axonal/unexcitable NCS. These patients are candidates for early tracheostomy.
